# Use of Therapeutic Pathogen Recognition Receptor Ligands for Osteo-Immunomodulation

**DOI:** 10.3390/ma14051119

**Published:** 2021-02-27

**Authors:** Paree Khokhani, Nada R. Rahmani, Anne Kok, F. Cumhur Öner, Jacqueline Alblas, Harrie Weinans, Moyo C. Kruyt, Michiel Croes

**Affiliations:** 1 Department of Orthopedics, University Medical Center Utrecht, 3584 CX Utrecht, The Netherlands; p.k.khokhani@umcutrecht.nl (P.K.); N.R.Rahmani@umcutrecht.nl (N.R.R.); a.k.kok3@students.uu.nl (A.K.); F.C.Oner@umcutrecht.nl (F.C.Ö.); j.alblas@umcutrecht.nl (J.A.); h.h.weinans@umcutrecht.nl (H.W.); M.C.Kruyt@umcutrecht.nl (M.C.K.); 2 Department of Biomechanical Engineering, Technical University Delft, 2628 CD Delft, The Netherlands

**Keywords:** multifunctional coatings, adjuvant, osteoimmunology, osteoblast, osteoclast, pathogen-recognition receptors, pathogen-associated molecular patterns

## Abstract

Therapeutic pathogen recognition receptor (PRR) ligands are reaching clinical practice following their ability to skew the immune response in a specific direction. We investigated the effects of various therapeutic PRR ligands on bone cell differentiation and inflammation. Following stimulation, alkaline phosphatase (ALP) activity (Day 10), osteocalcin, osteonectin expression (Day 14), and calcium deposition (Day 21) were quantified in bone marrow-derived human mesenchymal stem cells (hMSCs). The osteoclastogenic response was determined by measuring tartrate-resistant acid phosphate (TRAP) activity in human monocytes. TNF-α, IL-6, IL-8, and IL-10 expressions were measured by enzyme-linked immunosorbent assay as an indicator of the ligands’ inflammatory properties. We found that nucleic acid-based ligands Poly(I:C) and CpG ODN C increased early ALP activity in hMSCs by 4-fold without affecting osteoclast formation. These ligands did not enhance expression of the other, late osteogenic markers. MPLA, Curdlan, and Pam3CSK4 did not affect osteogenic differentiation, but inhibited TRAP activity in monocytes, which was associated with increased expression of all measured cytokines. Nucleic acid-based ligands are identified as the most promising osteo-immunomodulators, as they favor early osteogenic differentiation without inducing an exaggerated immune-cell mediated response or interfering in osteoclastogenesis and thus can be potentially harnessed for multifunctional coatings for bone biomaterials.

## 1. Introduction

Various off-the-shelf synthetic bone substitutes, such as biodegradable ceramics and polymers, are readily available in the clinic for use in orthopedic interventions [1,2,3]. Although the performance of certain synthetic replacements are even reported to be non-inferior to autograft in applications such as spinal fusion, the success rate is not optimal [4,5]. A challenge remains in their limited osteoinductive capacity, namely the ability to induce the differentiation of bone progenitor cells, during the critical early weeks following implantation [3,6,7]. To enhance the efficacy of synthetic bone biomaterials, one approach is the development of coatings that align with the biological process of bone formation.

Since the immune and skeletal systems are closely entangled, strategies are being explored to modulate the local host immune environment in favor of bone formation [6,7]. Referring to fracture healing as a model for efficient bone regeneration, the early inflammatory phase is proven to be a vital step in osteogenesis. During this initial phase, pro-inflammatory cytokines including TNF-α, IL-6, IL-8, IL-17 and anti-inflammatory cytokines such as IL-10 and IL-4 are being released at the site of injury and subsequently, recruit and differentiate progenitor cells towards the osteogenic lineage [8,9]. A balanced inflammatory reaction is key, as a sustained inflammatory response will mitigate the bone regenerative process [10,11,12]. Since microbial stimuli were found recently to be involved in osteo-immunomodulation, it is thought that the induction and transcription of pro-osteogenic cytokines can also result from the activation of pattern recognition receptor (PRR) signaling cascades present in immune or bone cells [11,13,14]. Indeed, the specific targeting of PRRs with ligands derived from intact or fragmented bacterial cell wall components has been demonstrated to induce local and transient inflammation that results in enhanced bone formation in vivo [15,16,17].

It is currently unknown which PRR ligands lead to the most optimal bone response, and which class of PRR ligands have highest clinical merit. The realization that PRR-targeting ligands can selectively initiate and propagate immune responses has provoked the pharmaceutical industry to develop synthetic immunological adjuvants that resemble microbial cell wall components or their nucleic acids, but with higher clinical merit due to high purity and stability, lower toxicity, and ability to resist rapid degradation in vivo [18,19,20]. This class of agents has shown success in the pre-clinical and clinical testing phase as vaccine adjuvants to enhance the adaptive immune response by antigen presentation or as stand-alone therapeutic agents to suppress or enhance the inflammatory response depending on the specific need, e.g., treatment of infections, the suppression of autoimmune responses, and the stimulation of anti-tumor immunity [21]. In the context of orthopedic application, it is yet unknown if the immune modulating capacity of synthetic therapeutic PRR ligands can be employed to recruit and dictate the fate of the various cells involved in the bone healing process. 

Mesenchymal stem cells (MSCs) are the progenitors of osteoblasts and therefore crucial target cells in nearly all clinical scenario’s necessitating bone regeneration [22,23]. MSCs express a multitude of PRRs that are thought to regulate their proliferation, differentiation and inflammatory properties [24,25,26]. Until now, the bone-forming capacity of PRR ligands has been explored in vivo mainly in rodents, with inconsistent responses in comparison to human cells [27]. These inconsistencies are likely related to the difference in the expression of PRRs in different species and the generally lower sensitivity of rodents to microbial stimuli [24,25]. To better predict the possible clinical reaction, further investigation is required to explore the response of human MSCs (hMSCs) towards therapeutic PRR ligand stimulation.

Apart from hMSCs, the local activity of osteoclasts is also important for bone regenerative strategies. Osteoclasts derive from the hematopoietic cell line and are considered early responders to PRR ligands after in vivo delivery [26,28]. Local depletion of osteoclasts or their precursors during early inflammation is suggested to impair the onset of new bone formation in biomaterials [28,29]. Therefore, potential therapeutic PRR ligands used for orthopedic settings should not have inhibitory properties towards osteoclast formation during the early phases of inflammation.

In this study we investigate the potential use of synthetically developed therapeutic PRR ligands as immunomodulators for bone regeneration. Different classes of therapeutic PRR ligands are evaluated for their effect on osteogenic differentiation of hMSCs, osteoclast formation and cytokine expression by both hMSCs and monocytes. 

## 2. Materials and Methods 

### 2.1. Study Design

A set of therapeutic PRR ligands (Table 1) was evaluated for their effects on osteogenic differentiation, osteoclast formation, and pro-inflammatory activity in human cells. Bone-marrow derived hMSCs were stimulated with the PRR ligands in dexamethasone-based osteogenic differentiation medium to identify possible modulatory effects on osteogenic differentiation. Moreover, hMSCs were stimulated with PRR ligands in expansion medium to discriminate between possible pro-osteogenic effect independent of osteogenic stimuli, namely osteoinduction. As a marker of early osteogenic differentiation, cells were assessed for the day 10 activity in alkaline phosphatase (ALP), an enzyme secreted by cells of the early osteoblast lineage and that plays a role in matrix calcification. As markers of late osteogenic differentiation, the day 14 expression of osteonectin and osteocalcin—non-collagenous proteins that regulate biological mineralization process—and day 21 biological mineralization were measured [27,30,31].

Next, PRR ligands were investigated for their modulatory effects on monocyte-derived osteoclast differentiation. We investigated the effects of PRR stimulation on day 6 tartrate-resistant acid phosphatase (TRAP) activity, an enzyme secreted by resorptive osteoclasts, in the presence of macrophage colony stimulating factor (M-CSF) and receptor activator of NF-κB ligand (RANKL), as the presence of both of these factors is a prerequisite for the differentiation of human monocytes into osteoclasts [26].

As the changes in local inflammatory milieu are thought to underlie the effects of PRR immunomodulation on osteoblast and osteoclast differentiation [32,33,34], the effects of PRR ligand stimulation were studied on the cytokine production by hMSCs and monocytes. The levels of pro-inflammatory (TNF-α, IL-6, IL-8) and anti-inflammatory (IL-10) cytokines were measured after 24 h stimulation, as they provide a general depiction of the degree of inflammation induced by the different PRR ligands.

### 2.2. Reagents

Poly(I:C) high molecular weight, CpG oligodeoxynucleotide (ODN) type C (M362), Resiquimod, Pam3CSK4, Monophosphoryl Lipid A (MPLA), Curdlan (Beta-1,3-glucan from *Alcaligenes faecalis*), and Murabutide were obtained from Invivogen (San Diego, CA, USA). These mediators were selected for their ability to activate different cell-surface and intracellular PRRs (Table 1). Their final concentrations were based on the ability to induce TNF-α production in human monocytes (Resiquimod, Pam3CSK4, MPLA Curdlan, and Murabutide) (Supplementary Figure S1) or the manufacturer’s data sheet (Poly(I:C) and CpG ODN). Recombinant human M-CSF and recombinant human RANKL were purchased from Peprotech (London, UK).

### 2.3. Cell Sources and Culture Conditions

Human material was obtained in accordance with the Declaration of Helsinki, with the approval of the local medical ethical committee (University Medical Center Utrecht, Utrecht, The Netherlands) under the protocols METC 08-001/K and METC 07-125/C, and with the written consent of the participants. Bone marrow was harvested from the vertebrae of female patients aged 15–30 years (*n* = 7), diagnosed with idiopathic scoliosis. These cell sources were selected because of the young donor age and the minimal probability of interfering factors like systemic diseases, co-morbidities, or use of medication.

hMSCs were isolated and cryopreserved as described in detail previously, as this method of MSC isolation yields multipotent cells as shown by standard differentiation assays along osteogenic, adipogenic, and chondrogenic lineages [35]. Cells below passage 7 were used for the experiments. Human peripheral blood from healthy volunteers aged 25–40 years (4 females, 1 male) was obtained from the Mini Donor Service (University Medical Center Utrecht, Utrecht, The Netherlands) in heparinized tubes. The mononuclear cell fraction was isolated by density centrifugation using Ficoll–Paque, followed by monocyte separation using positive CD14 magnetic-activated cell sorting (MACS), according to the manufacturer’s instructions (Miltenyi Biotec, Bergisch-Gladbach, Germany). MSC expansion medium consisted of α-MEM (Invitrogen, Carlsbad, CA, USA) with 10% (*v*/*v*) heat-inactivated fetal bovine serum (FBS, Hyclone CSG0412, GE Healthcare Life Sciences), 100 units/mL penicillin and 100 μg/mL streptomycin (Gibco), and 0.2 mM L-ascorbic-acid-2-phosphate (Sigma, St Louis, MO, USA). Monocyte medium consisted of RPMI (Thermo Fisher Scientific, Waltham, MA, USA) supplemented with 10% (*v*/*v*) heat-inactivated FBS and 100 units/mL penicillin and 100 μg/mL streptomycin. All cell culture experiments were performed at 37 °C in a humidified atmosphere containing 5% CO_2_.

### 2.4. hMSC Osteogenic Differentiation

To test the effect of PRR ligands on early and late osteogenic differentiation, hMSCs were seeded at a density of 15,000 cells/cm^2^ in a 96-well plate or 24 well-plate in technical triplicates, and cultured in MSC expansion medium. Upon 100% confluency, cells were cultured in expansion medium or osteogenic differentiation medium (expansion medium supplemented with 10 mM β-glycerophosphate and 10 nM dexamethasone), in absence or presence of PRR ligands (final concentrations in Table 1). The medium and PRR ligands were refreshed twice a week for 10 days (early analyses), 14 days and 21 days (late analyses). 

For ALP activity quantification, cells were lysed in 0.2% (*v*/*v*) Triton X-100/PBS for 30 min. ALP activity was measured by the conversion of the p-nitrophenyl phosphate liquid substrate system (pH = 9.6) (SigmaFast p-nitrophenyl phosphate tablets, Sigma-Aldrich). The absorbance was measured at 405 nm and corrected at 655 nm (Bio-Rad, Hercules, CA, USA). The cell lysate was also used to determine the DNA content with the Quant-It PicoGreen kit (Invitrogen, Carlsbad, CA, USA), according to the manufacturer’s instructions. The ALP/DNA was normalized for the control not receiving any PRR ligands. 

Osteocalcin (*n* = 3 MSC donors) and osteonectin (*n* = 2 MSC donors) expression were visualized using immunocytochemical staining. At day 14, cells were fixed in 4% (*w*/*v*) paraformaldehyde and permeabilized in 0.2% (*v*/*v*) Triton X-100/PBS. The cells were treated with 5% (*w*/*v*) bovine serum albumin (BSA)/PBS for 30 min in order to block aspecific binding of the antibody. Samples were incubated for 1 h at room temperature with 10 μg/mL mouse monoclonal antibody recognizing human osteocalcin (clone OCG4, Enzo Life Sciences, Zandhoven, Belgium) or 10 μg/mL anti-human osteonectin (AON-1, DSHB, Iowa City, IA, USA). The monoclonal mouse IgG_1_ antibody was used as isotype-matched control Ab. This was followed by 1 h incubation with 10 μg/mL goat-anti-mouse polyclonal antibody conjugated to Alexa Fluor 488 (Invitrogen). After washing with PBS, nuclei of the cells were stained with 1 μg/mL 4′,6-diamidino-2-phenylindole (DAPI) for 10 min. The staining was visualized using a fluorescence microscope (Thunder, Leica microsystems, Wetzlar, Germany). Quantification was done by measuring the mean intensity of the binary image (area of the image = 0.6 mm^2^) obtained from experiments done in duplicates per donor using ImageJ Freeware version 1.53e software (National Institutes of Health, Bethesda, MD, USA). 

To assess and quantify the matrix mineralization after 21 days, samples were incubated with 0.2% Alizarin Red S (ARS) for 60 min (pH = 4.2, Sigma) and examined using light microscopy. In addition, Alizarin Red was extracted from the monolayer of the cells by incubating in 10% (*w*/*v*) cetylpyridinium dissolved in 10 mM sodium di-phosphate buffer solution (pH = 7.2) (Sigma Aldrich) for 60 min. Absorbance was measured at 595 nm and corrected at 655 nm. The amount of calcium deposited in each well (experiments done in triplicates) was then quantified using the standard curve obtained by dissolving known concentration of ARS and considering 2 mol of Ca^2+^/mol of dye in solution.

### 2.5. Osteoclast Differentiation Assay

CD14+ monocytes (*n* = 3–4 donors) were seeded at a density of 500,000 cells/cm^2^ in a 96-well plate in technical triplicates and cultured in osteoclast differentiation medium, consisting of α-MEM supplemented with 10% (*v*/*v*) heat-inactivated FBS, 100 units/mL penicillin and 100 μg/mL streptomycin, 25 ng/mL M-CSF and 50 ng/mL RANKL. As a negative control for osteoclast differentiation, macrophage differentiation was induced using M-CSF medium in absence of RANKL. The effects of the PRR ligands were studied in osteoclast differentiation medium at a final concentration as mentioned in the figure legend. Culture was performed for a total of 6 days, with a medium/PRR ligand change on day 3. For TRAP staining, cells were fixed in 4% (*w*/*v*) paraformaldehyde and incubated for 20 min with 50 mM tartaric acid in 0.2 M acetate buffer (pH 5.0). Subsequently, 0.5 mg/mL naphtol AS-MX phosphate (Sigma-Aldrich) and 1.1 mg/mL fast red TR salt (Sigma-Aldrich) were added to the buffer and incubated for another 10 min at 37 °C. The samples were imaged on the IX53 Inverted Microscope (Olympus, Tokyo, Japan). Osteoclasts were defined as TRAP-positive cells (pink/red) with 3 or more nuclei [32] and were counted by a blinded observer (MC) in 4 predefined regions of interest (ROI) with a total surface area of 0.6 mm^2^. The osteoclast counts were normalized to the controls receiving only M-CSF and RANKL.

### 2.6. Cytokine Expression

hMSCs were seeded at a density of 62,500 cells/cm^2^ in MSC expansion medium in 96-well plates in technical triplicates. Monocytes were seeded at a density of 240,000 cells/cm^2^ in monocyte medium in 96-well plates in technical duplicates. Cells were stimulated for 24 h with PRR ligands according to the final concentrations mentioned in the figure legend and the supernatant was stored at −20 °C for cytokine determination. The concentrations of TNF-α, IL-6, IL-8, and IL-10 were measured using commercially available ELISA kits (Duoset, R&D Systems, Minneapolis, MN, USA), according to the manufacturer’s instructions. The results were normalized to the non-stimulated control.

### 2.7. Statistical Analysis

Data were tested for a normal Gaussian distribution using the Shapiro–Wilk normality test. Repeated measures ANOVA with Dunnett’s post hoc correction or repeated measures mixed model with Sidak’s post hoc correction were performed in SPSS (V24, IBM, Armonk, NY, USA using *p* < 0.05 as a threshold for significance. All data are presented as mean ± standard deviation, with the group sizes indicated in the figure legends.

## 3. Results 

### 3.1. Effect of PRR Ligands on hMSC Osteogenic Differentiation 

**Figure 1:**
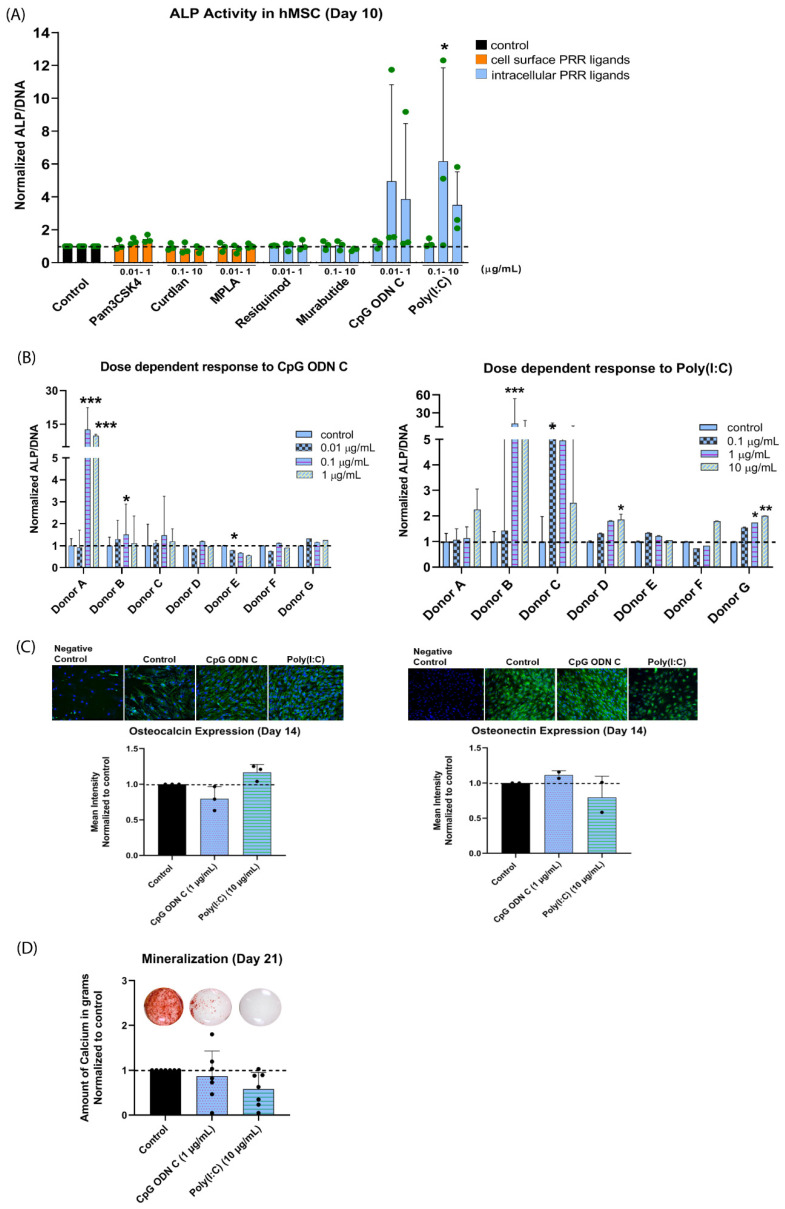
Effect of PRR ligands on human mesenchymal stem cells (hMSCs) osteogenic differentiation. (**A**) Alkaline phosphatase (ALP) activity in hMSCs after stimulation with PRR ligands in osteogenic differentiation medium for 10 days. hMSCs were stimulated with a concentration range of each PRR ligand (Table 1). ALP activity was corrected for DNA content of the cells and normalized to the control in osteogenic medium. Results show the mean ± standard deviation for 3 MSC donors. (**B**) ALP activity for 7 individual MSC donors after stimulation with CpG ODN C or Poly(I:C). Results show the mean ± standard deviation of the technical triplicate performed per donor. (**C**) Immunocytochemical staining for intracellular osteocalcin (representative for 3 MSC donors) and osteonectin (green) (representative for 2 MSC donors) was performed in hMSCs after stimulation with CpG ODN C (1 µg/mL) and Poly(I:C) (10 µg/mL) in osteogenic medium for 14 days. The staining was compared to the isotype control (negative control) to confirm the specificity of the positive signal. Cell nuclei were stained with DAPI (blue). Scale bar: 200 µm. Mean intensity of the images was calculated using ImageJ and normalized to the control in osteogenic medium. Results show the mean ± standard deviation. (**D**) Alizarin Red S staining was performed after stimulation with CpG ODN C (1 µg/mL) and Poly(I:C) (10 µg/mL) for 21 days (representative for 7 MSC donors). Total amount of calcium deposited per well was quantified and normalized to the control in osteogenic medium. Results show the mean ± standard deviation for 7 MSC donors. Significance was tested using repeated-measures ANOVA with Sidak’s post hoc test for multiple comparisons. * *p* < 0.05, ** *p* < 0.01, *** *p* < 0.001.

None of the cell surface PRR ligands affected the early osteogenic differentiation in terms of the ALP activity in hMSCs as compared to the control (Figure 1A). A significant 5-fold increase in ALP activity at day 10 was observed in the group stimulated with Poly(I:C), and a similar trend in increased ALP activity was observed in the group stimulated with CpG ODN C. On closer investigation of the responses per individual donor, it was observed that the effects of Poly(I:C) and CpG ODN C were both donor- and dose-dependent (Figure 1B). More specifically, only 4/7 donors were responsive to PRR stimulation in terms of significantly enhanced ALP activity. Poly(I:C) and CpG ODN C had a synergistic interaction with the osteogenic differentiation medium. No effects of these PRR ligands were observed when culturing the cells in expansion medium (Supplementary Figure S2), suggesting a lack of any osteoinductive effect. 

In contrast to the findings regarding the early osteogenic marker ALP, groups stimulated with Poly(I:C) and CpG ODN C did not show an increased response to the late osteogenic differentiation markers, i.e., osteocalcin and osteonectin expression at day 14 (Figure 1C) and biological mineralization at day 21 (Figure 1D). An inhibitory effect of Poly(I:C) in 5/7 donors and CpG ODN C in 4/7 donors was even seen in terms of biological mineralization. Less variation was observed in their protein expression among the donors (Supplementary Figure S3).

### 3.2. Effect of PRR Ligands on Cytokine Expression of hMSCs 

**Figure 2:**
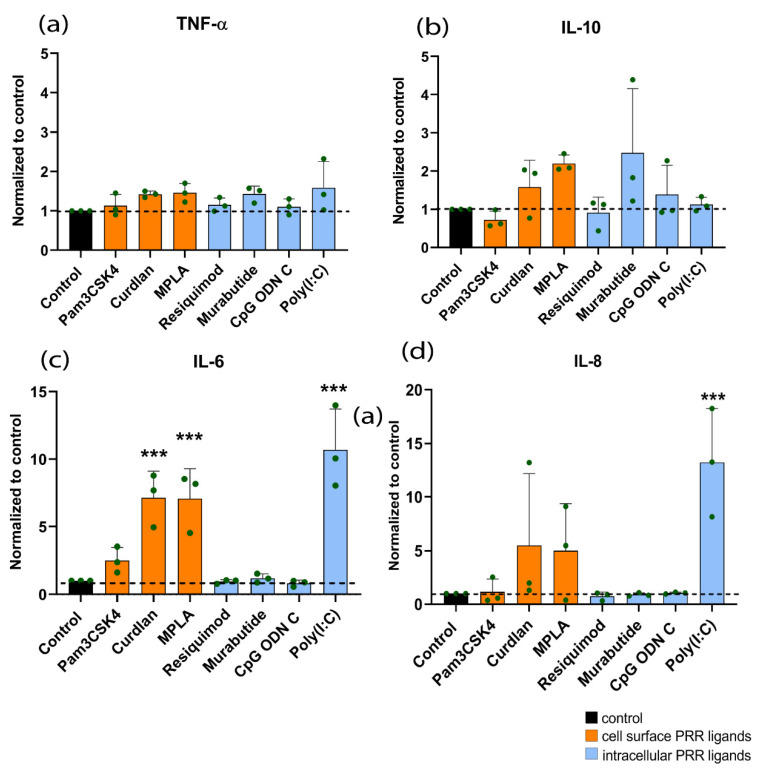
Cytokine production by hMSCs after stimulation with PRR ligands for 24 h. Cytokines measured were (**a**) TNF-α, (**b**) IL-10, (**c**) IL-6, and (**d**) IL-8. Concentrations used are the following: Pam3CSK4 (0.1 µg/mL), Curdlan (1 µg/mL), MPLA (10 µg/mL), Resiquimod (0.1 µg/mL), Murabutide (10 µg/mL), CpG ODN C (1 µg/mL), and Poly(I:C) (1 µg/mL). All results shown here are represented as mean ± standard deviation (*n* = 3 donors) and normalized to the control. Absolute values of the controls can be found in supplementary Table S1. Significance was tested using repeated-measures ANOVA with Sidak’s post hoc test for multiple comparisons. *** *p* < 0.001.

In hMSCs, generally low production of TNF-α and IL-10 was observed in comparison to the production of IL-6 and IL-8 (Supplementary Table S1). As compared to the non-stimulated control, a significantly increased production of IL-6 was observed following stimulation with Curdlan (7-fold), MPLA (7-fold), or Poly(I:C) (12-fold) (Figure 2c). Poly(I:C) stimulation resulted in a significantly higher production of IL-8 (15-fold) as compared to the control. A trend towards increased production of IL-8 was seen following treatment with the cell surface PRR ligands.

### 3.3. Effect of PRR Ligands on Human Osteoclast Differentiation 

**Figure 3:**
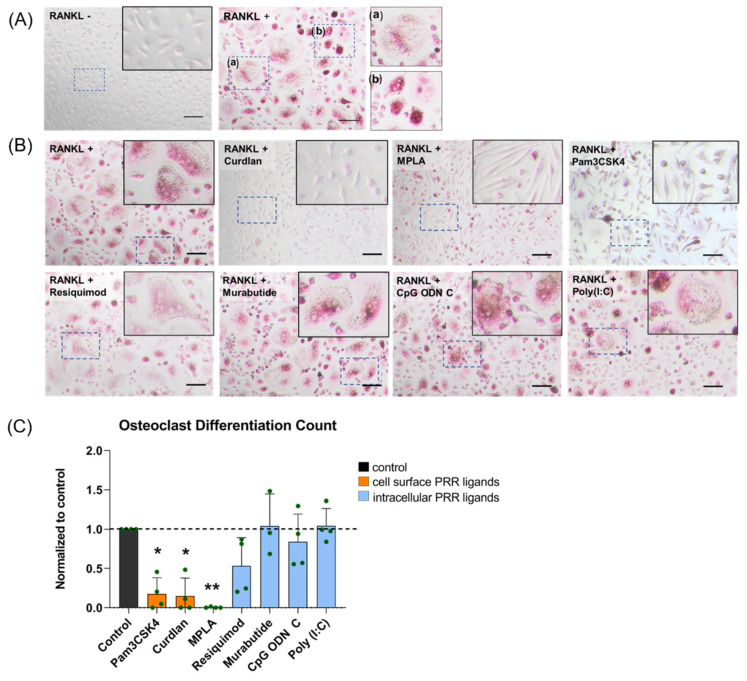
Effect of PRR ligands on osteoclast formation. (**A**) Tartrate-resistant acid phosphate (TRAP) staining performed on human monocytes cultured for 6 days +/- receptor activator of NF-κB ligand (RANKL). Osteoclasts are shown as TRAP-positive (red/pink) cells with ≥ 3 nuclei. (**a**) and (**b**) show variations in the shape and size of osteoclasts. (**B**) Representative images of monocytes stimulated with PRR ligands. Concentrations used are the following: Pam3CSK4 (0.1 µg/mL), Curdlan (1 µg/mL), Monophosphoryl Lipid A (MPLA) (10 µg/mL), Resiquimod (0.1 µg/mL), Murabutide (10 µg/mL), CpG ODN C (1 µg/mL), and Poly(I:C) (1 µg/mL). Scale bars correspond to 50 µm. (**C**) Osteoclast counts are represented as mean ± standard deviation (*n* = 3–4 donors) and normalized to the control. Significance was tested using a repeated-measures mixed model approach with Sidak’s post hoc test for multiple comparisons. * *p* < 0.05, ** *p* < 0.005.

It was confirmed that the combination of M-CSF and RANKL in the osteoclastogenic medium led to the differentiation of monocytes into TRAP-positive multinucleated osteoclasts. In comparison, monocytes maintained their mono-nucleated phenotype in absence of RANKL (Figure 3A). Cell-surface PRR ligands had strong effect as compared to intracellular PRR ligands on the day 6 TRAP activity in osteoclastogenic medium. A significant 75% decrease in TRAP activity was observed following stimulation with the cell surface PRR ligands Pam3CSK4 or Curdlan, while MPLA completely inhibited the TRAP activity. On the other hand, stimulation with the intracellular PRR ligands Resiquimod, Murabutide, CpG ODN C, or Poly(I:C) did not affect osteoclast differentiation (Figure 3B,C).

### 3.4. Effect of PRR Ligands on Cytokine Expression of Human Monocytes

**Figure 4:**
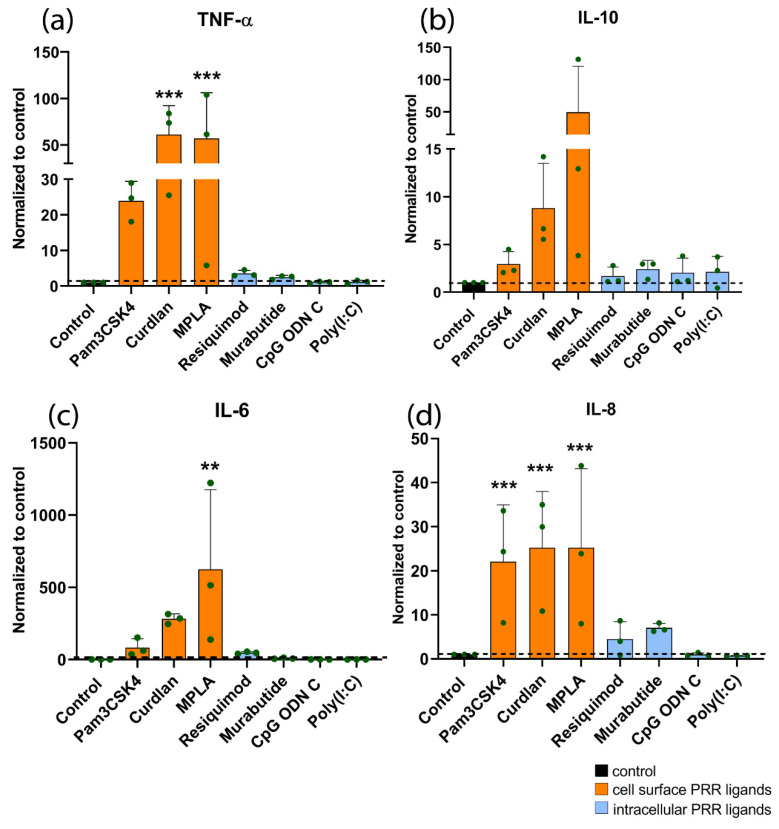
Cytokine production by human monocytes upon stimulation with PRR ligands for 24 h. Cytokines measured were (**a**) TNF-α, (**b**) IL-10, (**c**) IL-6, and (**d**) IL-8. Concentrations used are the following: Pam3CSKA (0.1 µg/mL), Curdlan (1 µg/mL), MPLA (10 µg/mL), Resiquimod (0.1 µg/mL), Murabutide (10 µg/mL), CpG ODN C (1 µg/mL), and Poly(I:C) (1 µg/mL). Results are represented as mean ± standard deviation (*n* = 3 donors) and normalized to the control. Absolute values of the controls can be found in supplementary Table S2. Normality of the data was tested using the Shapiro–Wilk test. Significance was tested using repeated-measures ANOVA with Sidak’s post hoc test for multiple comparisons. ** *p* < 0.01, *** *p* < 0.001.

Cell surface PRR stimulation in monocytes resulted in increased levels of cytokine production, whereas the intracellular PRR ligands had almost no effect. A significant increase in TNF-α production was observed following stimulation with Pam3CSK4 (20-fold), Curdlan (50-fold) or MPLA (50-fold) (Figure 4a). IL-6 production increased even 500-fold after MPLA treatment (Figure 4c). IL-8 production increased following stimulation with Pam3CSK4 (20-fold), Curdlan (25-fold), or MPLA (25-fold) (Figure 4d). Only a trend towards increased IL-10 production was seen following stimulation with cell surface PRR ligands (Figure 4b). Resiquimod, Murabutide, CpG ODN C, and Poly(I:C) had no significant effects on the cytokine production. 

## 4. Discussion

The current study identifies nucleic acid-based PRR ligands as the most promising osteo-immunomodulators. Poly(I:C) and CpG ODN C comprise of synthetic microbial RNA and DNA, respectively, and are currently being investigated in clinical trials to be used as clinical adjuvants in asthma therapy and anti-tumor therapies [23,36]. Upon recognition by the intracellular receptors TLR3 and TLR9, Poly(I:C) and CpG ODN C induce the activation of the NF-κB and TIR domain-containing adapter-inducing interferon β (TRIF) signaling pathways, which results in the secretion of mostly Interferon (IFN)-α, IL-6, and IL-10 in immune cells [36,37]. In our current work, it was found that Poly(I:C) and CpG ODN C strongly enhance the hMSC early osteogenic differentiation capabilities. In line with previous studies for selective pro-inflammatory cytokines, Poly(I:C), and CpG ODN C also acted in synergy with the osteogenic inducer dexamethasone [9,31]. It can be inferred from the current results that PRR ligands are capable of inducing pro-osteogenic cytokines in hMSCs favorable for osteogenesis. It was observed that the specific micro-environment induced by Poly(I:C), characterized by high IL-6 and IL-8 production in hMSC, could in part underlie the enhanced osteogenic differentiation seen, as these cytokines have demonstrated pro-osteogenic effects [38,39]. In agreement with recent studies, upon continuous stimulation with Poly(I:C), hMSCs may switch to a pro-inflammatory phenotype [40]. Although CpG ODN C treatment did not promote the production of any of the tested cytokines in hMSCs, it is likely that CpG ODN C stimulation may favor their production of TRIF-related cytokines similar as in immune cells [36]. Hence, in the future, it will be of importance to investigate the production of type I IFNs in the supernatant to get a better insight into the pathways leading to enhanced osteogenic differentiation. Considering the response of human monocytes derived cells to be most important sign of acute inflammation, absence of pro-inflammatory cytokines in human monocytes upon stimulation with these nucleic acids indicates a lack of immune cell mediated inflammation in vitro [11]. However, a strong inflammatory environment induced by the MSC may contribute and modulate the immune response in vivo. In contrast to other reports [31,41], TNF-α was found not to be a critical regulator of inflammation-induced osteogenesis in hMSCs. Whereas TNF-α expression is increased in immune cells after PRR stimulation [42], the low levels of TNF-α secreted by hMSCs after PRR stimulation has been explained by an absence of TNF-α enhancer complexes needed for their efficient TNF-α gene expression [40]. As a strong MSC donor dependency was seen in the changes in ALP activity in response to Poly(I:C) and CpG ODN C, it is of interest to regard the large differences in responsiveness to PRR ligands between human individuals [43,44,45] and establish a possible relationship between inter-individual differences in TLR expression by MSCs and the strength of the osteogenic responses.

Notably, there was an obvious discrepancy between the effects of CpG ODN C and Poly(I:C) on early and late osteogenic differentiation. Opposing the observed increase in ALP activity, Poly(I:C) and CpG ODN C did not increase the expression of late osteogenic markers, that is, osteocalcin, osteonectin, and matrix mineralization. In vitro, ALP activity in hMSCs is a far superior predictor of in vivo bone formation as compared to the late osteogenic markers osteocalcin and osteonectin [46]. Moreover, although biological mineralization is considered as functional end point of differentiation assays, there is evidence that ALP activity does not necessarily correlate with the ability of the cells to mineralize, and it is imperative to investigate the expression of Collagen type 1 and Bone sialoprotein along with biological mineralization [47]. Our in vitro findings are only partially predictive for in vivo bone formation, reason why the use of in vivo models is warranted to validate and verify whether nucleic acid-based ligands are indeed effective osteo-immunomodulators, considering that osteogenesis can only be determined in vivo.

To further determine the final role of nucleic acid-based PRR ligands in osteogenic differentiation and their optimal delivery method, it can be hypothesized that timing effects (i.e., short or continuous stimulation) may differently affect the expression of late bone markers. With this respect, shorter stimulation with CpG ODN C and Poly(I:C) may lead to more optimal upregulation of late osteogenic markers as compared to continuous stimulation to mimic the in vivo acute inflammatory response, as shown by many previous findings [48,49]. Moreover, given that innate immune cells mediate PRR ligand recognition and indirectly lead to cytokine-mediated regulation of hMSC functionality, the investigation of these PRR ligands in a co-culture setup comprising immune cells and hMSCs would provide additional insight into immune-mediated osteogenesis [50].

Considering that a normal osteoclast activity is a prerequisite for the onset of new bone formation around biomaterials [28,29], it was an important finding that, in addition to their pro-osteogenic effects, Poly(I:C) and CpG ODN C did not impair osteoclast formation. Although it was found that therapeutic PRR ligands could also profoundly affect human osteoclast formation, the outcome depends strongly on the cellular target [51,52,53]. Our study shows that in general, cell-surface PRR ligands (Curdlan, MPLA, and Pam3CSK4) markedly impaired osteoclast formation, whereas intracellular PRR-targeting ligands (Resiquimod, Murabutide, CpG ODN C, Poly(I:C)) did not influence osteoclastogenesis. In agreement, human monocytes have been reported to show higher expression of cell surface-bound TLR2 and TLR4 compared to intracellular TLR3, TLR7, and TLR9 [54], although the expression of these PRRs can also change in response to different stimuli [48,55].

Following stimulation with the former PRR ligands, a negative association existed between the expression of NF-κB-related pro-inflammatory cytokines produced by monocytes and RANKL-mediated osteoclast formation. While PRR activation and subsequent pro-inflammatory cytokine induction is known to mediate osteoclast formation through the NF-κB signaling pathway [43,44], it reportedly leads to varying outcomes depending on the maturation stage of the osteoclast precursor cell [49,50,56]. Early myeloid precursors not yet primed with RANKL maintain their phagocytic phenotype when exposed to PRR ligands [48,57,58]. On the other hand, PRR stimulation of committed myeloid precursor cells further increases the differentiation of myeloid cells into osteoclasts and maintains their survival [44,51]. In the current study, the monocytes were simultaneously exposed to PRR ligands and RANKL from the start of culture, as this is thought to best represent the acute in vivo setting after biomaterial implantation [59,60]. As RANKL is also generated by osteoblasts under the stimulation of pro-inflammatory mediators [32], future studies investigating the effects of PRR ligands towards osteoblast-mediated osteoclast formation would also be of interest.

With the current novel findings, several future strategies could lead to improved bone biomaterials for clinical use. The successful application of PRR ligands ideally establishes a balanced local innate immune response without leading to sustained or systemic reactions [33,52]. Following this premise, the delivery of nucleic acid-based PRR ligands in the form of nano-particles or other delivery vehicles could be formulated to more effectively target hMSCs and the bone formation process around biodegradable bone substitutes, since they could otherwise lead to suboptimal effectiveness or cause systemic toxicities [53,56]. Various coating techniques like layer-by-layer or direct immobilization of the nucleic acids have already been employed to modify biomaterial surfaces for the controlled release and/or the intracellular delivery of nucleic acids [57,58]. Moreover, many biological effects of nucleic acid-based PRR ligands could potentially lead to their multi-purpose use in orthopedic biomaterials at high risk of bacterial colonization [61,62]. CpG ODN pretreatment confers protection against different types of infection, involving changes in both innate and adaptive host immune cells [63]. Consequently, local pretreatment with CpG ODN has protective effects against *S. aureus* infection in a rat bone infection model [64]. At the same time, the immune protective effect of CpG ODN follows the premise that it enhances bacterial phagocytosis and intracellular killing by various cell types, including osteoblasts [65,66,67]. As opposed to CpG ODN, Poly(I:C) is a booster of the antiviral response. Since viral infections are known to increase the susceptibility to bacterial infections [68], it is questionable whether Poly(I:C) would have protective effects against bacteria commonly found in orthopedic infections. To illustrate, although Poly(I:C) is anti-infective towards Gram-negative bacteria [65], contrarily, mice pretreated with Poly(I:C) are more susceptible to Methicillin-resistant *Staphylococcus aureus* infection [69].

## 5. Conclusions

Therapeutic PRR ligands have immunomodulatory effects that exceed their current clinical indication, having different effects on bone-forming and bone-resorbing cell types. Most notably, the nucleic acid-based PRR ligands Poly(I:C) and CpG ODN C may have potential to be applied as immunomodulators of bone regeneration. As favorable characteristics to support new bone formation, these PRR ligands had a direct stimulatory effect on the early differentiation of hMSCs into the osteogenic lineage but without interfering with osteoclast formation. Since late osteogenic markers were not upregulated in response to these PRR ligands, further investigation and verification is necessary to determine the final role of nucleic acid-based PRR ligands in osteogenic differentiation and their optimal delivery strategy. The cytokine expression profiles of human MSCs and monocytes indicated that the early pro-osteogenic effects of Poly(I:C) and CpG ODN C were associated with a mild pro-inflammatory response. Since therapeutic PRR ligands have demonstrated anti-infective properties, it should be established whether nucleic acid-based ligands could be harnessed for multifunctional coatings that may enhance the bone forming capacity of synthetic biomaterial in orthopedic application, while mitigating the risk of orthopedic infections.

## Figures and Tables

**Table 1:** Overview of investigated pathogen recognition receptor (PRR) ligands and working concentrations.

Therapeutic PRR Ligand	Receptor	Natural Ligand	Concentration
Pam3CSK4	TLR1/2 ^a^	Bacterial lipoproteins	0.01–1 μg/mL
Curdlan	Dectin-1 ^a^	n/a	0.1–10 μg/mL
MPLA	TLR4 ^a^	Gram-negative bacterial Lipid A	0.01–1 μg/mL
Resiquimod	TLR7/8 ^b^	Microbial single-stranded RNA	0.01–1 μg/mL
Murabutide	NOD2 ^b^	Bacterial peptidoglycan	0.1–10 μg/mL
CpG ODN C	TLR9 ^b^	Microbial DNA	0.01–1 μg/mL
Poly(I:C)	TLR3 ^b^	Microbial double-stranded RNA	0.1–10 μg/mL

^a^ Cell-surface, ^b^ intracellular, TLR = toll-like receptor, NOD2 = nucleotide-binding oligomerization domain-2.

## Data Availability

The data that support the findings of this study are available from the corresponding author upon reasonable request.

## Supplementary Materials

The following are available online at https://www.mdpi.com/1996-1944/14/5/1119/s1. Figure S1: TNF-α production by human monocytes upon stimulation with PRR ligands for 24 h, Figure S2: Effect of PRR ligands on hMSCs on day 10 ALP activity in absence of dexamethasone. Figure S3: Effect of Poly(I:C) and CpG ODN C ligands on hMSCs on day 14 osteocalcin (*n* = 3) and osteonectin (*n* = 2) expression. Table S1: Absolute values in cytokine expression (pg/mL) by hMSCs, expressed as mean ± SD for each donor, Table S2: Absolute values in cytokine expression (pg/mL) by human monocytes, expressed as mean ± SD for each donor.
