# Supplementary material for: Use of Therapeutic Pathogen Recognition Receptor Ligands for Osteo-Immunomodulation
Source: Materials (Basel). 2021 Feb 27;14(5):1119. doi: 10.3390/ma14051119 (PMC7957819; doi:10.3390/ma14051119)
Supplement: Supplementary file 1 [file materials-14-01119-s001.pdf]

materials MDPI

Supplementary data

Use of Therapeutic Pathogen Recognition Receptor Ligands for Osteo-Immunomodulation

Paree Khokhani [1,†], Nada R. Rahmani [1,†], Anne Kok [1], F. Cumhur Öner [1], Jacqueline Alblas [1], Harrie Weinans [1,2], Moyo C. Kruyt [1] and Michiel Croes [1,*]

[1] Department of Orthopedics, University Medical Center Utrecht, Utrecht, The Netherlands; p.k.khokhani@umcutrecht.nl (P.K.); N.R.Rahmani@umcutrecht.nl (N.R.R.); a.k.kok3@students.uu.nl (A.K.); F.C.Oner@umcutrecht.nl (F.C.Ö.); j.alblas@umcutrecht.nl (J.A.); h.h.weinans@umcutrecht.nl (H.W.); M.C.Kruyt@umcutrecht.nl (M.C.K.)
[2] Department of Biomechanical Engineering, Technical University Delft, Delft, The Netherlands
* Correspondance: m.croes@umcutrecht.nl; Tel.: +31-887-556-97
† Authors contributed equally to the work.

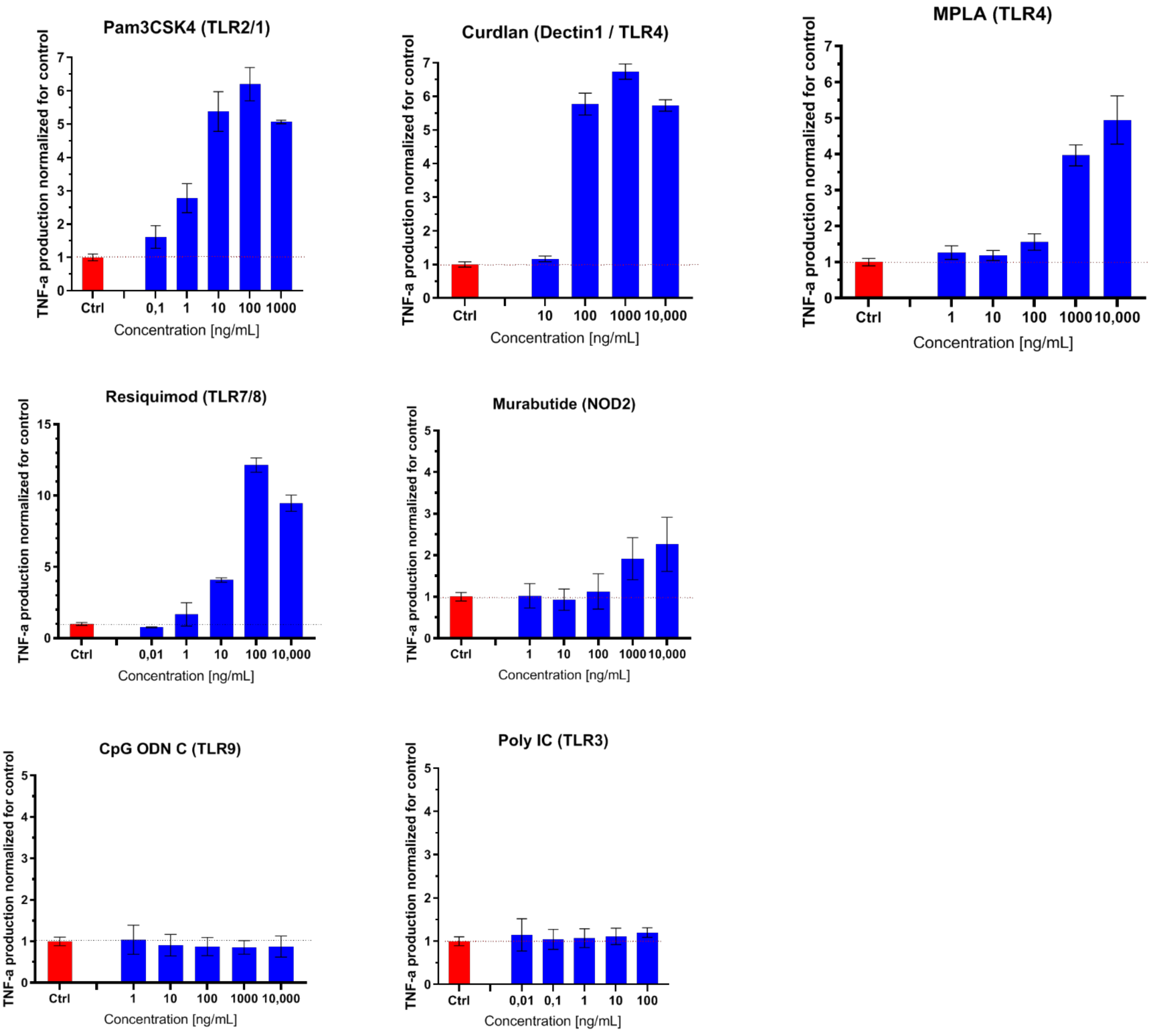

Figure S1. TNF-α production by human monocytes upon stimulation with PRR ligands for 24 hours (concentrations used in Table 1). Results are represented as mean ± standard deviation (n = 2 donors) and normalized to the control.

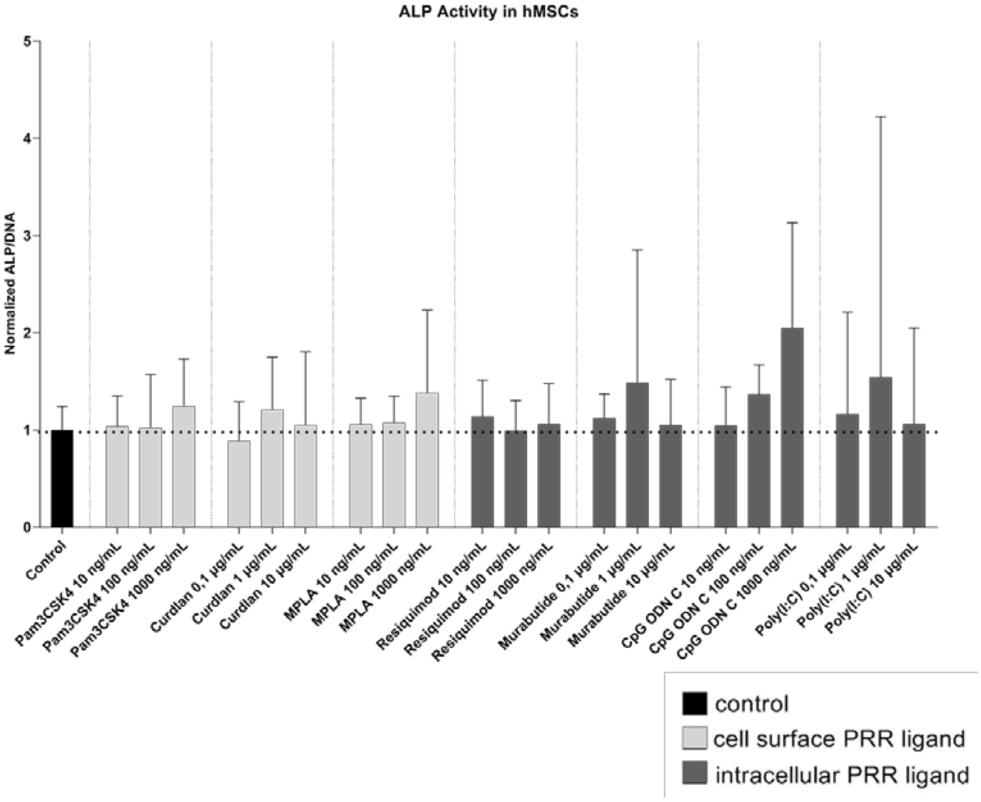

Figure S2. Effect of PRR ligands on hMSCs on day 10 ALP activity in absence of dexamethasone. Results are represented as mean ± standard deviation (n = 3 donors) and normalized to the control.

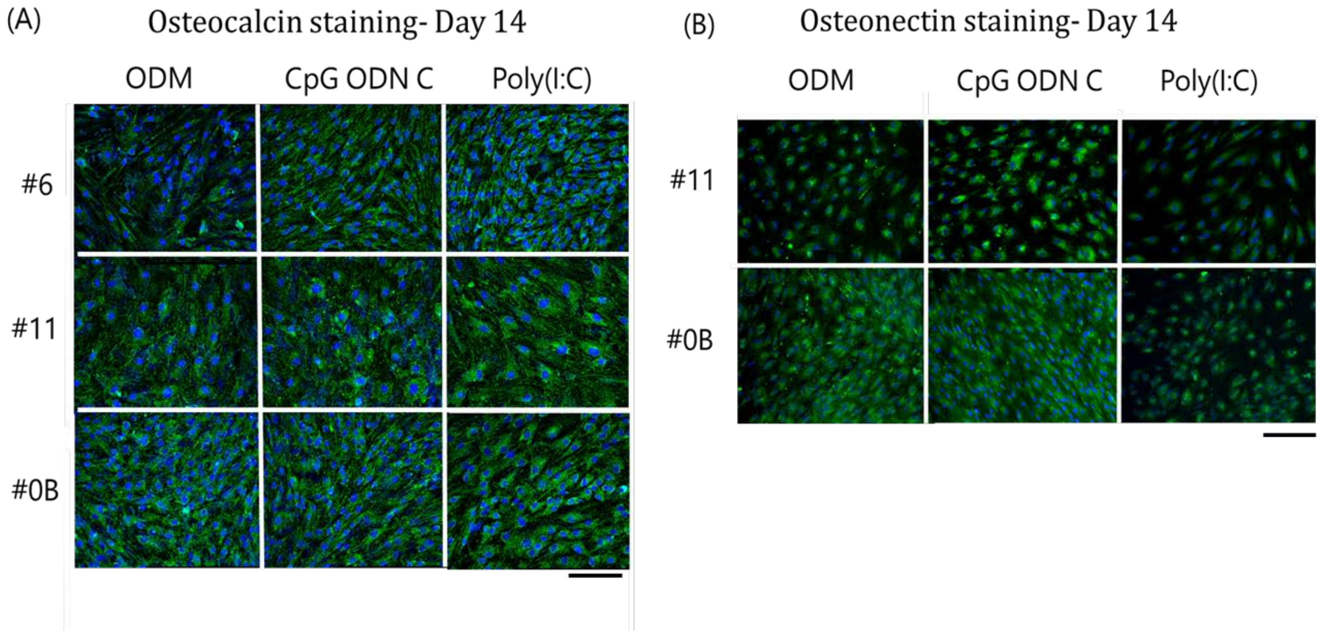

Figure S3. Effect of Poly(I:C) and CpG ODN C ligands on hMSCs on (A) day 14 osteocalcin (n = 3) and (B) osteonectin (n = 2) expression. Results are represented as mean ± standard deviation and normalized to the control (ODM) containing osteogenic medium. Scale bar = 200 µm.

Table S1. Absolute values in cytokine expression (pg/mL) by hMSCs, expressed as mean ± SD for each donor.

	**TNF-α**	**IL-10**	**IL-6**	**IL-8**
**Donor A**	17.70 ± 0.20	29.08 ± 21.06	1546.49 ± 59.58	2713.38 ± 225.14
**Donor B**	30.76 ± 3.41	73.52 ± 38.37	1497.58 ± 99.33	2776.52 ± 28.05
**Donor C**	32.42 ± 2.16	65.86 ± 8.00	1142.78 ± 83.51	2856.84 ± 41.84

Table S2. Absolute values in cytokine expression (pg/mL) by human monocytes, expressed as mean ± SD for each donor.

	**TNF- α**	**IL-10**	**IL-6**	**IL-8**
**Donor A**	27.15 ± 0.00*	11.379 ± 0.00*	9.38 ± 0.00*	1207.63 ± 0.00*
**Donor B**	29.04 ± 0.48	9.14 ± 0.85	9.38 ± 0.00*	1193.14 ± 539.23
**Donor C**	66.59 ± 7.72	42.27 ± 3.24	36.31 ± 27.56	4582.08 ± 2696.00

* The lowest concentration in the standard curve was used for calculations when absorbance values were below that of the lowest concentration in the standard curve.
